# Lactate as a myokine and exerkine: drivers and signals of physiology and metabolism

**DOI:** 10.1152/japplphysiol.00497.2022

**Published:** 2023-01-12

**Authors:** George A. Brooks, Adam D. Osmond, Jose A. Arevalo, Justin J. Duong, Casey C. Curl, Diana D. Moreno-Santillan, Robert G. Leija

**Affiliations:** Exercise Physiology Laboratory, Department of Integrative Biology, University of California, Berkeley, California, United States

**Keywords:** cardiopulmonary regulation, glucose paradox, lactate shuttle, lactylation, metabolic signaling

## Abstract

No longer viewed as a metabolic waste product and cause of muscle fatigue, a contemporary view incorporates the roles of lactate in metabolism, sensing and signaling in normal as well as pathophysiological conditions. Lactate exists in millimolar concentrations in muscle, blood, and other tissues and can rise more than an order of magnitude as the result of increased production and clearance limitations. Lactate exerts its powerful driver-like influence by mass action, redox change, allosteric binding, and other mechanisms described in this article. Depending on the condition, such as during rest and exercise, following carbohydrate nutrition, injury, or pathology, lactate can serve as a myokine or exerkine with autocrine-, paracrine-, and endocrine-like functions that have important basic and translational implications. For instance, lactate signaling is: involved in reproductive biology, fueling the heart, muscle adaptation, and brain executive function, growth and development, and a treatment for inflammatory conditions. Lactate also works with many other mechanisms and factors in controlling cardiac output and pulmonary ventilation during exercise. Ironically, lactate can be disruptive of normal processes such as insulin secretion when insertion of lactate transporters into pancreatic β-cell membranes is not suppressed, and in carcinogenesis when factors that suppress carcinogenesis are inhibited, whereas factors that promote carcinogenesis are upregulated. Lactate signaling is important in areas of intermediary metabolism, redox biology, mitochondrial biogenesis, neurobiology, gut physiology, appetite regulation, nutrition, and overall health and vigor. The various roles of lactate as a myokine and exerkine are reviewed.

**NEW & NOTEWORTHY** Lactate sensing and signaling is a relatively new and rapidly changing field. As a physiological signal lactate works both independently and in concert with other signals. Lactate operates via covalent binding and canonical signaling, redox change, and lactylation of DNA. Lactate can also serve as an element of feedback loops in cardiopulmonary regulation. From conception through aging lactate is not the only a myokine or exerkine, but it certainly deserves consideration as a physiological signal.

## Graphical abstract

**Figure:**
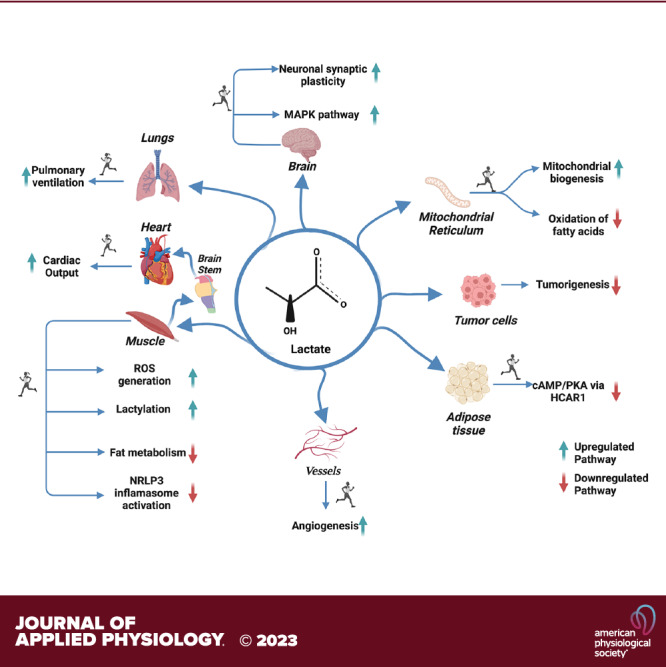

## INTRODUCTION

Although lactate has traditionally been viewed as a metabolic waste product and cause of muscle fatigue, there has been a revolution in understanding its role in normal and pathophysiological conditions (1–9). Lactate is formed under fully aerobic conditions during postprandial rest and exercise (4, 10–12). The roles of lactate as a preferred energy substrate and gluconeogenic precursor have previously been reviewed (2, 10, 12–14). Hence, the many roles of lactate as a signaling molecule and driver of biochemical and physiological processes are presented here.

Lactate shuttles and signals within and among cells, organs, and tissues. As indicated later, the roles of lactate in metabolism and exercise performance have received much attention.^1^ However, recognition of the regulatory attributes of lactate is more recent (2). In contrast to more commonly recognized myokine signaling moieties such as IL-6 that exist in pico- or nano-molar concentrations (15), lactate exists in millimolar concentrations in muscle, blood, and other tissues. As well, the dynamic range of lactate concentration is more than an order of magnitude under normal physiological and pathological conditions.

**Figure 1.**
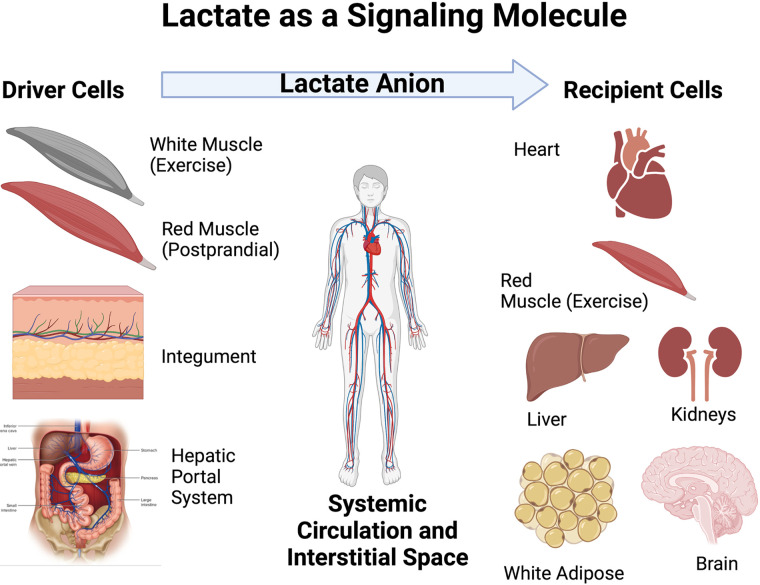
Illustration of the roles of driver and recipient cells in lactate shuttle signaling. Lactate fluxes from sites of production and high concentration in driver cell compartments and tissues to sites of lower concentration in recipient disposal sites. Depending on metabolic conditions some sites can switch from driver to recipient cells. Examples of switching are several and include initial lactate release from muscle beds at the onset of exercise to uptake by the same muscle bed as blood flow and oxygenation increase to meet metabolic demands. At that time, other tissues such as the integument become lactate shuttle drivers. Another example occurs after carbohydrate nutrition when red skeletal muscle takes up glucose and releases lactate as part of the “postprandial lactate shuttle.” Seen from the perspective of Fig. 1, lactate shuttling provides for fuel energy carbon exchange and metabolic signaling. Figure modified from Ref. 4. Recreated with BioRender.com.

Myokines and exerkines are substances that have autocrine-, paracrine-, and endocrine-like functions when released from muscles. Lactate serves as a myokine when produced in resting muscles, and as an exerkine when produced during exercise, in the integument and working muscles. Aspects of lactate production, removal, and signaling have important basic and translational implications. For instance, lactate fuels sperm motility, supports embryonic development (16, 17), is the most rapidly assimilated and oxidized sports drink component (18), and has potential to be a treatment for the brain following trauma (19–22). Because its production is increased during exercise, some regard lactate to be an exerkine (15). However, lactate holds even more importance as a myokine that operates continuously, during rest, after a meal, and during exercise and recovery (3, 4). Based on our own independent research and review of the literature, we assert that lactate signaling is important in areas of intermediary metabolism, redox biology, mitochondrial biogenesis, cardiovascular and pulmonary regulation, genomics, neurobiology, gut physiology, appetite regulation, pathways of carbohydrate nutrient metabolism, and skeletal and overall body vigor and health. Indeed, while the role of lactate can be described as a myokine or exerkine, there is potential for the nomenclature to include a host of other, yet unnamed “-kines” representing major tissue sites of lactate turnover (e.g., integumentokine, enterokine, neurokine, hepatokine, spermatokine, phagokine, erythrokine, mitokine, etc.) (Table 1 and Fig. 1).

**Table 1.** Lactate as a signaling molecule: drivers, targets, messengers, and actions

Driver	Downstream Messenger/Action	Target Cell/Tissue	Biological Action	References
Contracting skeletal muscle	HCAR-1	Adipocytes tissue, neurons, and skeletal muscle	Inhibits lipolysis, inflammation suppression, muscle hypertrophy	(23–28)
Contracting skeletal muscle	CPT-2	Mitochondria	Inhibits fatty acid uptake and oxidation	(29)
Contracting skeletal muscle	Histone lactylation	DNA/nucleus	Post-transcription alterations	(30, 31)
Contracting skeletal muscle	PGC-1α	Metabolically active tissue	Stimulates mitochondrial biogenesis	(32–34)
Contracting skeletal muscle	IGF-1	Metabolically active tissue	Stimulates skeletal muscle hypertrophy	(25)
Contracting skeletal muscle	Sirtuins 1 and 3	Metabolically active tissue	Stimulates mitochondrial biogenesis	(32, 35, 36)
Contracting skeletal muscle	Allosteric binding?	Lyding cells	Increase testosterone	(37, 38)
Contracting skeletal muscle	BDNF	Dentate gyrus of the hippocampus	Stimulates neurogenesis	(39–43)
Contracting skeletal muscle	VEGF	Endothelial cells	Promotes angiogenesis	(44–46)
Contracting skeletal muscle	Olfr78	Carotid body	Stimulates pulmonary ventilation	(47)
Contracting skeletal muscle	Allosteric binding	Metaboreflex types III&IV sensory fibers	Stimulates pulmonary ventilation	(48)
Contracting skeletal muscle	Allosteric binding	Myoglobin	Increases deoxygenation	(49–51)
Gut	GLP-1	Intestinal L-cells	Stimulates insulin secretion	(52)
Gut	GPR132	Intestinal mucosa	Incretin secretion	(53)
Postprandial red muscle	Ghrelin	Hypothalamus	Suppression of appetite	(54, 55)
Contracting skeletal muscle	TGF-β2	Adipose tissue	Increased secretion of TGFβ-2, improved insulin sensitivity	(56)
Cancer cell	p62	Tumor stroma cells	Decreases autophagy/increase cancer cell proliferation	(57, 58)
Sodium lactate incubation	Histone deacetylase	CD8+ cells	Inhibited tumor growth	(59)
Contracting skeletal muscle/postprandial skeletal muscle	Allosteric binding, redox?	Liver & kidneys	Increased gluconeogenesis	(4, 60, 61)
Bone	Allosteric binding, redox?	Skeletal remodeling	Osteoclast activation	(62)

And finally, by way of introduction to this review, current understanding of lactate signaling and sensing largely falls within the realm of metabolism. This is because lactate signaling and sensing are consequences of production with outcomes and feedback control typical of physiological systems. Hence, it is difficult to strip lactate metabolism from a discussion of signaling and sensing. More subtle aspects of lactate signaling and sensing in the absence of large changes in lactate production will likely be discovered in the future. For instance, in reproduction biology, the timing of lactate signaling is important (17).

## HISTORIC BACKGROUND: LACTATE SIGNALING AMONG PRODUCER (DRIVER) AND CONSUMER (RECIPIENT) CELLS

To pioneer researchers (63, 64), lactate shuttling was not obvious because the rate of production and appearance in blood (Ra) equals disposal (Rd, rate of disposal from the blood) in most circumstances. To the pioneers, only conditions when blood lactate concentration rose or declined (i.e., Ra ≠ Rd) were observable. But, as required by chemistry, physics, and physiology (Fick’s law), solutes flux from high to lower concentrations, and back to a limited extent. Hence, in that context the metabolism of metabolites, such as lactate, can be understood. This means lactate production and release from “driver” cellular compartments, cells, tissues, and organs is counterbalanced by uptake and metabolic disposal elsewhere at “recipient” sites (Fig. 1). Necessarily, cell type, metabolic and dietary states, integument, cardiovascular, enterokine, lymphatic and hepatorenal systems are involved. In fairness to the pioneers, concepts of neuroendocrine, myokine or exerkine signaling had not yet been developed.

Initial “lactate shuttle” theory was based on simultaneous glucose and lactate flux measurements (65, 66), and lactate concentration differences in tissues of resting and exercising rats (67, 68). Hence the idea of lactate flux from fast, glycolytic to oxidative (69) fiber types was deduced (1, 12, 70). Subsequently, it became obvious that lactate released from working muscle beds was taken up and oxidized by the heart (71, 72). Moreover, implicit in the results was the understanding that similar phenomena occurred at rest when concentration gradients and turnover rates were much less compared with those during exercise (73, 74).

Understanding that tissue participation in lactate shuttling could change over time was foreshadowed in work of Welch and Stainsby (75) on dog muscles contracting in situ. Gastrocnemius-plantaris muscles released lactate at the onset of electrically induced contractions, but switched to net uptake as contractions continued. Hence, it was not surprising that the same phenomenon (Stainsby Effect) was seen in human muscles during continuous exercise (76, 77). In exercising men, the switch in muscle from net lactate release to uptake coincided with increases in blood flow and oxygen delivery to match metabolic demand (76). Subsequently, and perhaps more importantly, studies of human subjects led to recognition that resting and working human muscles simultaneously produced and consumed lactate, and that elevated blood lactate concentration (lactatemia) and high blood lactate turnover persisted during exercise when muscles switched from net release to uptake (76). This latter observation meant that some other tissue was the net producer, and hence the “driver” of circulating lactate availability. Although this facet of lactate shuttling is basically uninvestigated, it has been observed that under sympathetic stimulation, as occurs in exercise, glycogenolysis and glycolysis in the integument results in net lactate release (78). Beyond the integument, other organ sites of lactate production and net release into the circulation remain to be identified; inactive skeletal muscle (79), and the gluconeogenic liver and kidneys are probably not good candidates for lactate release (80) in exercising humans.

The initial “lactate shuttle” posited glycolytic to oxidative tissue lactate exchange (1). However, while less obvious, lactate shuttling is also apparent at rest when digestive, circulatory, musculoskeletal, hepatorenal, and probably lymphatic systems are involved. For instance, studies of postprandial glucose metabolism in animal models and humans show what has been termed as the “Glucose Paradox,” or “Indirect Pathway of Hepatic Glycogen Synthesis” (81). This concept recognizes that dietary glucose released into the hepatic portal vein initially bypasses the liver and goes to the periphery where glycolysis converts glucose to lactate that is subsequently released into the venous circulation and taken up from the arterial circulation by liver for glycogen synthesis. This, paradoxical, “Indirect” pathway is to be contrasted with the “Direct” pathway in which dietary glucose from the gut is taken up from the hepatic portal vein and converted to liver glycogen on first circulatory pass.

The initial concept of an Indirect Pathway of Hepatic Glycogen Synthesis was developed from studies on laboratory animals and has been replicated in human subjects showing both indirect and direct liver glycogen synthesis in healthy, postprandial humans. However, the balance of Indirect and Direct glucose conversion to hepatic glycogen appears to be species related. It has been confirmed in human subjects that glycolysis was the main initial postprandial fate of glucose that accounted for most of overall disposal while oxidation and storage accounted for the remainder. However, the majority of hepatic glycogen synthesis in postprandial humans (>73%) was formed via the Direct Pathway (82). In the near future, it should be possible to better understand how diet and other factors (e.g., hepatic glycogen content, sex, age, physical activity level, insulin action) influence the balance of direct versus indirect liver and muscle glycogen synthesis in men and women using deuterium- and ^13^C-labeled glucose and lactate tracers with magnetic resonance spectroscopy (MRS) of liver and skeletal muscle (83).

Although considered to be a homogeneous “organ system,” muscle is in fact a heterogeneous tissue containing different types of muscle fibers, circulatory and connective cells and tissues, motor nerve networks, and progenitor (satellite) cells among others (84). Skeletal muscle fiber types have different metabolic and contractile characteristics owing to differences in myosin isoform expression and densities of capillary and mitochondrial networks (69, 85, 86). Postural muscles (e.g., soleus, erector spinae) are alternatively termed Intermediate, (red slow oxidative), or Type I fibers. In many species, deep vastus and lateral gastrocnemius are bright red and termed Red or Type IIA fibers. In contrast white, fast twitch fibers are termed Type IIX (in humans) or IIB (in other mammals). Results of the earlier-cited studies on the Indirect Pathway of Hepatic Glycogen Synthesis are complemented by results of studies on dogs postfeeding showing greater postprandial perfusion and glucose uptake in muscles containing predominantly oxidative Type I and IIA fibers (87, 88). Thus, Types I and -IIA fibers are drivers of the “postprandial lactate shuttle,” whereas Types IIB and IIX fibers are drivers of cell-cell (fiber to fiber) lactate shuttling during moderate to hard intensity exercise with all fiber types contributing organ-organ (muscle to heart) during maximal lactate efforts (4). Lactate flux rates and tissue exchanges during exercise recovery are little studied, but oxidative tissue sites with high mitochondrial reticulum densities, liver, and kidneys likely playing major roles as splanchnic vasoconstriction are relaxed. Seemingly, knowledge that mild exercise during recovery from strenuous efforts helps clear lactatemia (89), studies of inter-organ lactate shuttling during exercise recovery might prove useful for developing protocols to reduce lactate accumulation by mild functional electrical stimulation (FES) in conditions such as sepsis (90).

In closing this section on the history of lactate biology, it is important to note that the ideas of lactate as the product of oxygen-limited metabolism and metabolic waste came into prominence because of the early history and preeminence of researchers, including two Nobel Laureates (A.V. Hill, Otto Meyerhof, and others of similar distinction (Rodolfo Margaria, David B. Dill). In retrospect, it is regrettable that the findings of another Nobel Laureate, Otto Warburg on tumor metabolism (91) were not more broadly interpreted because glycolysis leading to lactate production is now recognized to occur under fully aerobic conditions (3, 14). However, limitations in classical theory had negative effects on advancing the fields of lactate biology and its translation to clinical practice. Hopefully, this article will have an effect of opening the doors leading to a better understanding of the central role of lactate in physiological and metabolic regulation, signaling, and sensing. More expansive reviews of the history of lactate metabolism are available and recommended (3, 10, 14, 92).

## THE FORMS OF LACTATE SIGNALING

### Peroxisome Proliferator-Activated Receptor Gamma Coactivator-1 Alpha, Reactive Oxygen Species, and Related Signaling

The effect of repeated exercise bouts (i.e., endurance training) on stimulating mitochondrial biogenesis is a classic finding (93, 94). Among the multiple upstream regulators of mitochondrial biogenesis is lactate, which activates peroxisome proliferator-activated receptor gamma coactivator-1 alpha (PGC-1α) and generates reactive oxygen species (ROS). Incubation of C2C12 myocytes with lactate results in upregulation of hundreds of genes apparently mediated by PGC-1α and ROS (32–34). The effect of intermittent lactate exposure simulating exercise on myogenesis in cultured C2C12 myoblasts via ROS generation has been replicated (33). Moreover, in mice, repeated intraperitoneal injection of dichloroacetate (DCA), an inhibitor of lactate production, minimized increases in mRNA levels of citrate synthase, cytochrome oxidase (COx), and fatty acid translocase (FAT/CD36) induced by training (95). More recently, it has been discovered that histone lactylation affects the expression of many genes (30, 31), including those of skeletal muscle proteins (R.G. Leija, A.D. Osmond, J.A. Arevalo, J.J. Duong, and G.A. Brooks GA, unpublished observations.).

### Intermediary Metabolism

Muscle contractions and carbohydrate (CHO) nutrition influence numerous metabolic pathways; some pathways (e.g., muscle glycolysis and glycogenolysis) are activated, while others (e.g., fatty acid mobilization and oxidation) are inhibited (4). Lactate is often a major factor in determining outcomes of those pathways. Whether an individual is resting or exercising, fasted or postprandial, the inevitable products of glycolysis in muscles under fully aerobic conditions are lactate anions and hydrogen ions (3, 14). These downstream products of metabolism are exported from sites of production and are exchanged within the muscular interstitium, released into the venous effluent, and distributed to organs and tissues via systemic circulation. Lactate and proton releases are indirectly linked (11, 96), not equivalent, and have individual effects.

Previously termed a “lactormone” (2), lactate exists in millimolar (mM), not nano- or pico-molar concentrations as are other myokines (15). For example, arterial lactate concentration rises from ∼0.5 mM at rest to greater than 20 mM in arterial blood during hard exercise (97). Furthermore, lactate concentration in the venous effluent belies intramuscular production whereas arterial levels are less due to dilution as well as cardiac and pulmonary parenchyma metabolism (4, 98, 99). Via vascular conductance during exercise, lactate is an energy substrate for the heart, red skeletal muscle, brain, and liver (72) (Fig. 1).

### Redox Biology

**Figure 2.**
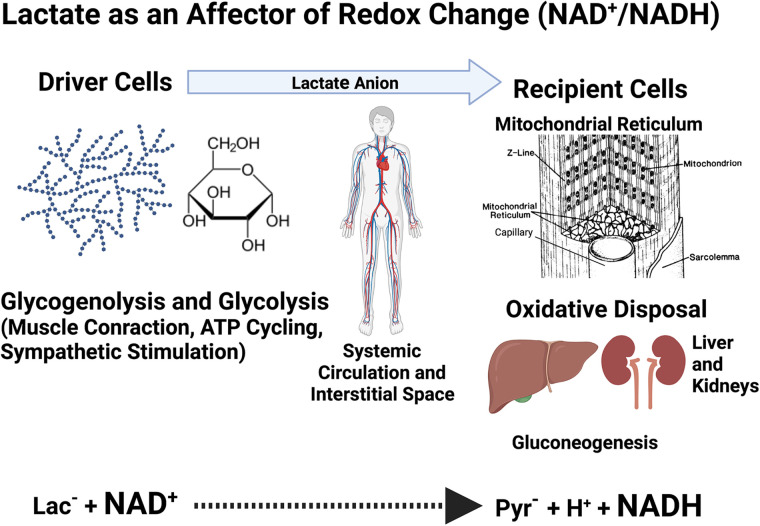
Illustration of the cellular redox exchange caused by lactate shuttling. At driver sites, lactate production results for reduction of pyruvate to lactate. However, at recipient sites oxidation of lactate to pyruvate occurs. Pyruvate reduction to lactate and subsequent oxidation of lactate to pyruvate result in millimolar changes in cellular NADH/NAD^+^ ratios. Among other forms of lactate signaling described in text or Fig. 3, changes in cell redox caused by lactate shuttling are most profound. Figure is a pictorial representation of data in Ref. 102, created with BioRender.com.

As determined from the venous effluent of working muscles (100), or muscle biopsies (101), the lactate/pyruvate ratio (L/P) in resting muscle (nominally 10) can rise an order of magnitude or more during exercise (100, 101). The change in L/P, a surrogate for the NADH/NAD^+^, reflects massive cytosolic redox changes in both producer and conversely, in consumer cells and tissues (Fig. 2). Lactate accumulation results in ROS production via enzymatic and spontaneous reactions (32, 103). Furthermore, glycolytic flux to lactate activates Sirtuins 1 (SIRT-1) and 3 (SIRT-3) via its effect on NAD^+^ levels. With few exceptions, these effects of lactate production on redox status at sites of production (i.e., driver cells) and disposal (i.e., recipient cells) (13), have not been widely recognized, e.g., (15).

### Allosteric Binding and Inhibition of Lipolysis

**Figure 3.**
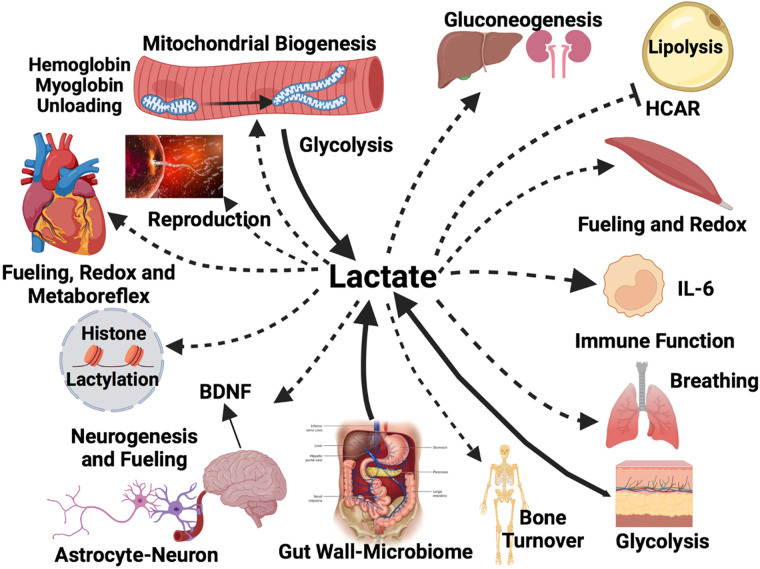
Illustration of diverse forms of intracellular lactate shuttling. Lactate producer (Driver) cells and tissues (broad solid lines and arrow heads) contributing to circulating lactate include contributions from the integument, gut, fast-glycolytic skeletal muscle, postprandial red skeletal muscle, and mixed skeletal muscle at the onset of exercise. Lactate consumer (Recipient) sites disposing of lactate (dashed lines and lesser arrow heads) include mitochondrial lactate oxidation in red and mixed skeletal muscle, the heart and brain during steady rate exercise. Also included are (dashed lines and lesser arrow heads) for lactate disposal via gluconeogenesis in the liver and kidneys, and for brain neurons (as part of the ANLS). Lactate-stimulated IL-6 release from monocytes and working muscle is an example of lactate-stimulated cytokine release. Whether drivers or recipients, all cells experience redox signaling effects. Signaling sites not involving carbon exchange or transformation include white adipose where lactate inhibits lipolysis via HCAR and CREB signaling, the heart when peripheral muscle lactate accumulation stimulates the metaboreflex with afferent signaling to the medullary cardiovascular center via Types III- and -IV sensory fibers which increases cardiac output, pulmonary ventilation via the carotid body olfactory receptor (Olfr78), the skeletal muscle where stimulates mitochondrial biogenesis via peroxisome proliferator-activated receptor gamma coactivator-1 alpha (PGC-1α), reactive oxygen species (ROS) and sirtuin activation. Further, lactate has the following actions: In working muscle lactate dissociates oxymyoglobin and blood oxyhemoglobin; in the brain lactate from the arterial circulation of glycolysis in astrocytes fuels neurons and participates in glutamatergic signaling as well as stimulates neurogenesis in the hippocampus and brain-derived neurotropic factor (BDNF) secretion. Moreover, lactatemia and tissue lactate accumulation have an epigenetic effect via lactylation of histones, and lactate has anti-inflammatory effects. Tissues involved starting top left and looking clockwise: skeletal muscle fibers, gluconeogenic organs the liver and kidneys, white adipose tissue, working red skeletal muscle, monocytes, the lungs, integument, skeleton, gut wall and microbiome, the brain, all nucleated cells containing DNA, the heart, ova, and sperm. Created with BioRender.com. Solid and dashed lines indicate flux directions, but not rates because typically lactate Ra = Rd in a steady state.

Initially identified as an orphan G protein-coupled receptor, GPR-81 has been renamed hydroxycarboxylic acid receptor 1 (HCAR-1) (23, 24). HCAR-1 is a lactate receptor that inhibits lipolysis via cAMP response element binding protein (CREB) activation in adipose and other diverse tissues (Fig. 3). Plasma free fatty acid concentrations fall during hard exercise in part because of the inhibition of lipolysis following the rise in circulating lactate (3). The effect of lactate signaling via HCAR-1 on lipolysis is little appreciated (15).

### Mitochondrial Energy Substrate Utilization

When activated muscle glycolysis and glycogenolysis result in the production of lactate and pyruvate with the L/P being 10 at rest, and rising more than an order of magnitude during moderate and greater intensity exercise (98, 101). Oxidation of the monocarboxylates yields acetyl-CoA and subsequently, via acetyl-CoA carboxylase, malonyl-CoA, a ligand that inhibits carnitine palmitoyltranferase 1 (CPT-1), and hence the uptake and oxidation of activated long-chain fatty acids (104). More recently, allosteric binding of lactate to cardiolipin has been associated with downregulation of CPT-2, further limiting mitochondrial uptake and oxidation of activated fatty acids (29). Thus, lactate is involved in downregulation of carbon flux at both initial and terminal ends of the pathway from fatty acid mobilization to oxidation. Rephrased, lactate markedly suppresses fat metabolism during exercise. However, during exercise recovery, lactate clearance has permissive effects on fatty acid mobilization and oxidation (56, 105, 106). Hence, exercise recovery is a time of lipid mobilization and oxidation.

### Mitochondrial Biogenesis

The mitochondrial reticulum, now characterized as the “energy grid of the cell” (107) provides the necessary fuels needed to handle various metabolic perturbations (108, 109). It is well documented that endurance exercise training and increased lactate turnover promote mitochondrial biogenesis (93, 110, 111) by increasing transcription and synthesis of mitochondrial proteins and their insertion into the mitochondrial reticulum (112). The metabolic stress of exercise raises lactate and AMP levels. The latter activates AMPK, an energy sensing molecule that supports maintenance of cellular energy homeostasis by numerous mechanisms including stimulation of mitochondrial biogenesis (113). Lactate acts as a major upstream signal of peroxisome proliferator-activated receptor gamma coactivator-1 alpha (PGC-1α), the master regulator of mitochondrial biogenesis (32, 114). Taken together, lactate, AMPK, ROS, PGC-1α, SIRT-1, and SIRT-3 play important roles in promoting mitochondrial biogenesis (32, 35, 36).

Although it is likely that lactate is involved in mitochondrial biogenesis as described earlier, it is also equally, or perhaps more likely that lactate is involved in the muscle hypertrophy of resistance training (25). As reviewed recently by Lawson et al., lactate works to stimulate muscle hypertrophy independent of and, in some ways, in concert with muscle tension. Powerful muscle contractions put the tissue under tension and simultaneously activate the glycolytic pathway leading to lactate production. One signaling pathway leading to muscle hypertrophy is lactate activation of insulin like growth factor 1 (IGF-1), downstream of which are protein kinase B (PKB) and mammalian target of rapamycin (mTOR). In synergy, lactate and muscle tension join in mTOR signaling of the ribosomal protein p7056K1, and subsequently ribosomal protein S6 (rpS6) that leads to increased muscle protein synthesis (MPS). A second mechanism by which lactate stimulates muscle hypertrophy is via HCAR binding and activation of the mitogen-activated protein kinase (MAPK) pathway that simulates satellite cell proliferation and growth. A third lactate effect is to inhibit myostatin and increase activity of folistatin that, again, stimulates satellite cell proliferation and growth. As well, lactate inhibits histone deacetylases (HDAC) leading to histone acetyl-transferase (HAT) activity increasing histone acetylation and lactylation and increasing gene expression that increase MPS (25).

Finally, on the subject of lactate-stimulated muscle hypertrophy, lactate may stimulate testosterone secretion. Not surprisingly, the rise in blood lactate following hard exercise accompanies increases in testosterone independent of changes in luteinizing hormone (LH). The apparent correlation may be explained by studies on isolated Leydig cells in which lactate stimulates testosterone production (37). Furthermore, dose-dependent increases of cAMP and testosterone production has been observed (38). Those results were interpreted to mean that lactate has a stimulatory effect on testosterone secretion via cAMP level modulation. Testosterone is considered an anabolic hormone, playing a primary role in activating mTOR, a major affecter of MPS.

To summarize this section, it is fair to reiterate that the roles for lactate in regulation of gene and protein synthesis regulation are becoming recognized (115).

### Vascular, Cardiac, and Pulmonary Regulation

It is well established that endurance exercise training promotes angiogenesis, a process mediated by growth factors such as vascular endothelial growth factor (VEGF) (44). Notably also, in wound healing and repair, lactate stimulates the release of VEGF and other growth factors to promote angiogenesis (45, 46). Furthermore, with regard to the cardiovascular system, it is recognized that lactate is the major fuel for the heart during exercise (71, 72, 116). Moreover, lactate increases mRNA levels of PGC-1α and COx expression in the heart (117). Perhaps most importantly, lactate accumulation in active muscle increases cardiac output by stimulating muscle metaboreceptors with afferent input to central cardiovascular regulatory centers via Types III and IV sensory fibers as part of the metaboreflex (48, 118). As well, it has been shown that lactate increases pulmonary ventilation during exercise via the carotid body olfactory receptor (Olfr78) in mice (47). Supporting data on Olfr78 functioning in humans is lacking.

In addition to working with many other mechanisms and factors increasing oxygen delivery by raising cardiac output, and maybe pulmonary ventilation, lactate participates in deoxygenation of hemoglobin at the tissue level (i.e., the Bohr effect) in which both hydrogen ions and lactate anions serve as competitive inhibitors of oxygen association with hemoglobin and myoglobin. Originally described by Hochachka et al. (119) as part of a unifying theory of hypoxia tolerance, and expanded upon by Clanton et al. (49, 50), the effect of lactate in promoting oxygen release from oxymyoglobin independent of hydrogen ion has recently been confirmed (51).

And finally, on the subject of the role of lactate in cardiopulmonary and cardiovascular medicine, we respectfully acknowledge existence of a large body of work on the anaerobic threshold (AT) (120, 121). That subject has been recently reviewed (92), but it is fair to state that while the inflection in circulating lactate during graded exercise was misinterpreted to signal the onset of tissue hypoxia, at no time did proponents of the AT suggest alternative signaling roles of lactate such as those enumerated here.

### Lactate and the Inflammasome: Is Lactate an Assailant, Defender, or Innocent Bystander?

A growing body of literature can be interpreted to mean that lactate is an upstream, physiological signal that, depending on the stress, and tissue, can act in an anti- or proinflammatory capacity, often mediated by downstream cytokines and other mechanisms.

#### Delayed onset muscle soreness.

Historically, muscle soreness following hard exercise has been attributed to lactate accumulation. However, lactate disposal is rapid, typically clearing in minutes after exercise while delayed onset muscle soreness (DOMS) peaks 24–48 h after hard exercise, long after lactate is cleared (122). Contrary to long-standing ideas in the etiology of DOMS, it may well be that lactate is anti-, not proinflammatory. For example, Hoque et al. (26) showed that lactate binding to HCAR-1 downregulates Toll-like receptor induction of the pyrin domain-containing protein 3 (NLRP3) inflammasome and production of IL1-β, via Arrestin β 2 (ARR-β2). Examples of HCAR-1 binding by lactate outside of exercise also supports the response in the inflammasome and is the mechanism by which lactate suppresses inflammation in patients with acute organ injury such as acute pancreatitis (26, 27), hepatitis (26), and sepsis (28). In addition, Chu et al. (123) found elevated levels of H3K17 lactylation in patients with sepsis compared with healthy volunteers, exhibiting this epigenetic modifier as an important biomarker. Overall, changes in lactate concentration sufficient to bind lactate to HCAR-1 and downregulate NLRP3 inflammasome are important examples of lactate functioning as a myokine and exerkine.

#### Chronic inflammation and autoimmunity.

Lactate is high (10 mM) in joints of patients with rheumatoid arthritis (124). In those spaces there occurs a positive feedback loop in which CD4+ T cells produce high levels of proinflammatory cytokine, IL‐17, while the anti-inflammatory capacity of CD8+ T cells is reduced, thus aggravating the inflammation (125). Similarly, in a mouse model of allergic asthma there occurs a proliferation of T cells with production of proinflammatory cytokines IL‐5, IL‐17, and IFN‐γ in airway mucosa (126). The proinflammatory response was inhibited by use of DCA an inhibitor of pyruvate dehydrogenase kinase (PDK), leading to pyruvate dehyrdrogenase (PDH) activation and redirecting glycolytic flux to oxidative disposal. However, experiments with the LDH blocker oxamate were not conducted. Hence, the apparent correlative findings implicating a role of lactate in proinflammatory responses illustrate the need for mechanistic explanations of why lactate concentration was elevated; was production elevated, or clearance is reduced, and what are the sequela by which lactate activates or suppresses inflammatory responses? Foreshadowing what the results might be, for the present it looks that while endogenously produced lactate might elicit proinflammatory responses, exogenously supplied lactate may have anti-inflammatory effects. Hence, a redox control mechanism may be implicated.

### Lactate Signaling, the Microbiome, and the Splanchnic Bed

Functional roles for the gut microbiome and its role in health and disease are currently of significant interest (127), particularly because of relationships between microbiota and the prevalence of chronic conditions such as insulin resistance and metabolic syndrome (128). Lactate appears in the gut by several mechanisms, including the consumption of probiotics (e.g., fermented foods) containing lactate and prebiotic, fiber-containing foods that promote fermentation and lactate production. In the colon, Lactobacillus, Bifidobacterium, and Firmicutes ferment fiber-containing carbohydrate foods to pyruvate and lactate. How lactate and other products of gut fermentation have systemic effects is a topic of investigation. However, one mechanism may be related to the presence of sodium-mediated monocarboxylate (lactate) transporters (sMCT) in intestinal mucosa (129, 130). Depending on concentration gradients, sMCT expression in the gut can either export lactate after a meal rich in fructose (131), glucose (4), or pre- or probiotics, or take up lactate after hard exercise that results in lactatemia.

For completeness on this section it is worth noting that some bacterial species produce racemic (l and d) lactate enantiomers, the d isoform being neurotoxic (132–134). Regrettably, d-lactatemia is often difficult to detect because many current technologies only detect the presence of the l isoform.

One mechanism by which gut lactate may affect systemic metabolism is through enteral signaling after eating, specifically by lactate stimulating sensory nerves associated with mesenteric lymphatic fluid (MLF) (Gregory W. Aponte, personal communication). Using a rodent model investigators in the Aponte laboratory and their collaborators have observed that after eating, glucagon-like peptide-1 (GLP-1) and glucose-dependent insulinotropic polypeptide (GIP) are secreted and induce the release of substance P (SP) that enhances insulin secretion (52). Like the actions of GLP-1 and GIP in their roles as incretins (i.e., substances that lower blood glucose levels by stimulation of insulin secretion), lactate also stimulates SP-containing afferent nerves associated with MLF, thus contributing to the control of blood glucose concentration after eating. With regard to the role of the secretion of incretins it would not be surprising that lactate signaling involves GPR132, which, like GPR81 (HCAR-1), signals through cAMP and CREB (53). As suggested previously, lactate release from the bowel into the systemic circulation via sMCTs with disposal elsewhere in the body indicates the presence of a “gut-soma lactate shuttle” (3). This area of lactate kinetics and signaling in promoting gut and systemic health begs for further investigation.

### The Intestinal Mucosa, Liver, and Hepatic-Portal Circulation

Classically, it has been understood that the liver and kidneys are the splanchnic sites of lactate disposal via gluconeogenesis for maintenance of glycemia (60), or hepatic glycogen synthesis (61). However, with realization of the “indirect pathway of hepatic glycogen synthesis” (81), and the “postprandial lactate shuttle” (4), a question now arises as to whether the liver, or splanchnic bed as a whole, can contribute lactate to the systemic circulation. Evidence for splanchnic lactate production is sparse, but is supportive.

In rats instrumented with indwelling portal vein catheters, a porto-peripheral lactate gradient was present after glucose ingestion, reflecting the production of lactate in or by the intestine (135). With regard to hepatic lactate production, lactate release from the liver under glucagon stimulation was not seen in dogs (136). These cross-species comparisons implicate the upper gastrointestinal (GI) tract as a site of lactate production.

Despite a dearth of direct (arterial-venous difference, a-v) information on splanchnic lactate production in humans, information from the sports nutrition field may be helpful. Using combinations of glucose, fructose, and lactate tracers to evaluate the use of oral carbohydrate energy sources in sports drinks investigators have observed carbon atoms from an orally ingested fructose tracer to appear in the systemic circulation as labeled lactate (131, 137). Hence, there is evidence for postprandial splanchnic lactate release in humans following the ingestion of one carbohydrate energy source, fructose. A similar phenomenon following ingestion of the disaccharide, sucrose (glucose + fructose) is likely (3).

### Hunger, Appetite, and Nutrition

In the context of overall factors affecting human health and nutrition that are released in response to changes in physiological status, perhaps no less important is the influence exerkines have on aspects of nutrition such as hunger and appetite.

The biochemistry behind hunger regulation is a complicated and active area of research. However, it is clear that the arcuate nucleus of the hypothalamus is the site of hunger regulation (138, 139). The gut hormone ghrelin is one of the hormones that informs the hypothalamic centers of body energy status (140, 141). The suppressive effect of hard exercise on appetite (142–144) is consistent with results that lactatemia acts via suppression of ghrelin secretion (54, 140). The ghrelin receptor [growth hormone secretagogue receptor (GHSR-1α)] is a G-protein coupled receptor expressed throughout both the stomach and GI tract. Recently, it was found that lactate, short chain fatty acids, and other bacterial excretions in the GI tract are able to attenuate ghrelin-mediated signaling through the GHSR-1α (55). Hence, in combination with lactate produced by gut microbiota, the heightened levels of blood lactate during exercise can enter the bowel via sMCTs and attenuate ghrelin receptor signaling, thus revealing how hard exercise attenuates hunger.

Another mechanism by which the lactatemia of exercise and illness may have a suppressive effect on appetite and hunger, and therefore obesity (3), is that lactate readily crosses the blood-brain barrier via monocarboxylate transporters (MCTs) and directly affects hypothalamic function (145). Initial results on brain tissues ex vivo are supported by results of studies using magnetic resonance spectroscopy (MRS) on healthy individuals (146). Anecdotally, hunger disappeared in studies on 12-h fasted men given exogenous lactate infusion (147). Also of note, athletes competing in 400–1,500 m runs that result in extraordinary lactatemia are seldom hungry immediately after hard training or competition.

And finally on the apparent linkages between lactatemia, appetite suppression, and resistance to obesity, based on studies on several mammalian species, including humans, it appears that lactate complexed with phenylalanine (Lac-Phe) downregulates appetite and prevents obesity (148). As recently reported, the production of Lac-Phe is catalyzed by the enzyme carnosine dipeptidase 2 (CNDP2) that is apparently substrate concentration driven and is expressed in macrophages, monocytes, and other immune and epithelial cells in diverse organs. The arcuate nucleus or other site of Lac-Phe action is yet to be determined. However, at this point it is probably appropriate to note that while the authors described sprint exercise blood lactate levels in excess of 25 mM, the corresponding Lac-Phe level approximated 200 nM, a 125-fold difference between lactate and Lac-Phe concentrations (their Fig. 5, *E* and *F*). In this purported signaling pathway, the driver molecule is apparent. Lactate in high physiological conditions likely complexes with (lactylates) many other biologically important substances including amino acids, proteins, and nucleic acids, vide infra.

### Lactate and the Brain

The brain demonstrates the capacity to oxidize lactate as an energy source (149–152). As part of glutamatergic signaling, astrocytes take up glucose from the blood and produce lactate to be shuttled to neurons that utilize lactate as the primary energy source in what is known as the “astrocyte-neuron lactate shuttle” (ANLS) (153, 154). In neurons, lactate signals by virtue of HCAR-1 binding (24), as well as redox signaling (3, 13). Importantly, in healthy humans, the lactatemia of exercise results in increased cerebral lactate uptake and improved executive function (39, 155). As well, using isotopic tracers, brain lactate uptake was undiminished in patients with traumatic brain injury (TBI) compared with healthy controls with over 90% of lactate uptake being oxidized in both groups (151, 152). Those results led to the idea of supporting recovery of TBI patients by exogenous l-lactate infusion (19).

Physical exercise leads to the release of brain-derived neurotropic factor (BDNF) (156) in the dentate gyrus of the hippocampus resulting in neurogenesis. More recently, studies of arterial-venous differences and cerebral blood flow measurements show that hard exercise leading to lactatemia results in cerebral lactate uptake followed by BDNF release (39). Furthermore, researchers have shown higher exercise intensity, eliciting higher blood lactate concentrations, increased cognitive function, independent of sex or BDNF polymorphisms (40). Importantly, utilizing exogenous lactate infusion into resting subjects, Schiffer et al. (41) showed the effect of lactate on brain BDNF release, thus demonstrating a mechanism dependent on lactate signaling as opposed to some other factor such as irisin (15). Several genes involved with neuronal synaptic plasticity, such as *Arc, Zif268, c-Fos, SRF*, and *BDNF* are upregulated in the presence of lactate in primary neurons of mice. The upregulation of these genes favors the development of long-term memory (LTM), via the activation of the *N*-methyl-d-aspartate receptor (*NMDAR*) and the Erk1/2 cascade, through an intracellular redox state change (42, 43). For BDNF, the expression is regulated by the silent information regulator 1 (STIR1) dependent induction of the *PGC1α/FNDC5* pathway (42).

In studies of NMDAR-dependent neuronal plasticity, a genome-wide transcriptional analysis detected a group of genes that are upregulated by exposure to lactate. Included are genes involved in the mitogen-activated protein kinase (*MAPK)* signaling pathway that plays a crucial role on cell proliferation (157). Lactate signaling and activation of the MAPK pathway is discussed later.

### Lactate Signaling of TGF-β2

An obvious contradiction in the literature involves the short- and long-term effects of exercise on lipid mobilization and oxidative disposal. As reviewed earlier, moderate to hard exercise results in lactatemia, crossover to CHO dependence (158), and inhibition of lipolysis during hard exercise in humans regardless of training state when HCAR-1 signaling is known to be activated (159, 160). In contrast are data on a mouse model indicating that lactate released during exercise caused transforming growth factor-β2 (TGF-β2) to be secreted from adipose tissue, which resulted in improved glucose tolerance (56). On the basis of their elegant work, the authors proposed a lactate-TGF-β2 signaling axis. Seemingly, these conflicts would be resolved by studies on humans showing that TGF-β signaling is responsible for increased lipid metabolism following exercise when crossover to lipid oxidation occurs (106, 158). If so, an important mechanism by which lactate affects the regulation of energy substrate partitioning during and after exercise would be revealed. Glycolysis and glycogenolysis during exercise produce lactate. Through HCAR-1, lactate first inhibits lipid mobilization and oxidation. Then, via TGF-β2 signaling, lactate sets into motion events giving rise to increased metabolic flexibility during exercise recovery after lactate is cleared (161).

The aforementioned paradox involving HCAR-1 and TGF-β2 antagonism appears to be one of several paradoxes surrounding lactatemia, exercise, and exercise training. Another noteworthy paradox is that the proinflammatory cytokine IL-6 released from working muscle may be the long sought “muscle factor” by which hepatic glucose release is matched to metabolic demand during exercise (162). Another paradoxical effect of lactate signaling stems from the multiple effects attributed to TGF-β2. Although TGF-β2 is involved in the purported lactate-TGF-β2 signaling axis (56), TGF-β2 is potentially injurious. For instance, disruption of the blood brain barrier (BBB) following injury results in TGF-β activation and stimulation of the Smad2 complex, which in turn leads to protein degradation via inhibition of AKT/mTOR pathway (163). TGF-β activation following disruption of the BBB illustrates that TGF-β may not always be a beneficial exerkine.

### The Rose Has Thorns: Lactate in Maladies Including the Emperor Cancer, and in Mimicking Glucose

Static measurements of lactate concentration indicate lactatemia is associated with severity of disease and poor prognosis (164). But is lactate accumulation the result of poor clearance, or is it an appropriate strain response to a stressor? In illnesses and injuries, the question is seldom asked and typically unanswered (28, 90). Nevertheless, inappropriate lactate signaling may be implicated in conditions as diverse as tumorigenesis and the regulation of insulin secretion during exercise.

#### Lactate in cancer.

Warburg and Minami (91) first described the metabolic phenotype characteristic of cancer cells. They noted high glucose uptake and excessive lactate formation in cancer cells even under fully oxygenated conditions; hence adoption of the term “Warburg Effect” (165), sometimes also inappropriately described as “aerobic glycolysis’ even though oxygen is neither a substrate for, nor a product of glycolysis. Still, while the high glucose uptake and lactate release phenotype remains a hallmark of cancer, there is no consensus on the meaning of the Warburg Effect. Initially, the excessive lactate formation of cancer cells and tumors led Warburg to propose that cancer was an injury to the cellular respiratory apparatus. However, cancer cells have mitochondria that are capable of respiring with lactate (166, 167). In contrast, many similarities between cancer and healthy exercise phenotypes have been described (168). Consequently, it was proposed that augmented lactate production (lactagenesis) initiated by gene mutations is the reason and purpose of the Warburg Effect and that dysregulated lactate metabolism and signaling are key elements in carcinogenesis (58). Support for the hypothesis of dysregulated lactate metabolism in carcinogenesis (3, 9, 168) is found in the results of recent experiments showing that lactate secreted from cancer cells into the stroma surrounding tumors downregulates p62 transcription in stromal cells through a mechanism involving redox change (i.e., the NAD^+^/NADH ratio, vide supra), which impairs poly(ADP-ribose)-polymerase 1 (PARP-1) activity. Subsequently, PARP-1 inhibition prevents the poly(ADP-ribosyl)ation of AP-1 transcription factors, c-FOS and c-JUN, which is an obligate step for p62 downregulation (57). Furthermore, it was shown that PARP inhibitors mimic lactate in the reduction of stromal p62 levels, as well as the subsequent stromal activation both in vitro and in vivo. These findings may give rise to a drug effective at inhibiting cancer-associated fibroblasts.

Lactate shuttling in tumors has led to serious attempts to repress tumorigenesis by blocking the release of lactate from highly glycolytic, glucose-consuming cells and those that respire lactate (169–173). Monocarboxylate transporters (MCTs) are bi-directional symporters facilitating movement of protons and lactate anions down concentration gradients (174). Although MCTs are ubiquitous and scaffolded in plasma membranes of most cells, including cancer cells, erythrocytes, and cells in the heart, muscle, and brain (175–177), blocking MCTs has been considered a possible pharmaceutical target in cancer research. However, the lack of a drug to target cancer cells has been a problem (178). As the quest to find cancer-specific MCT blockers has been unsuccessful as of yet, others are looking for alternative approaches to blocking lactate shuttling in tumors and cancer, such as by limiting the expression of CD147, the scaffold for MCT insertion into cell membranes (vide supra) (179–182), knocking down lactate dehydrogenase (LDH) expression (183), by preventing the reduction of stromal cell p62 levels (57), or by interfering with lactate signaling by silencing HCAR-1 (9). Yet again, the ubiquitous presence of proteins engendering lactate signaling requires that pharmacological blockers target tumors, not the host.

#### Lactate, lactate dehydrogenase and the glycolytic phenotype in cancer.

Originally believed to reside exclusively in the cytoplasm, LDH is now widely accepted as part of the mitochondrial reticulum and is annotated in the MitoCarta (184) and MitoMiner (185).^2^ Furthermore, using immunocoprecipitation and colocalization technologies mitochondrial LDH can be found in and visualized in muscle histological sections (186) as well as in cultured myocytes (187). Most recently, excised bands identifying LDH in isolated muscle mitochondrial preparations subjected to proteomic analysis confirm that the bands are LDH. In the cytosol, the equilibrium constant (*K*eq ≈1,000) pushes the conversion of pyruvate to lactate. Necessarily then, for lactate to become the major fuel for cell respiration, mitochondrial LDH is necessary for lactate oxidation to pyruvate (188–191). Therefore, targeting cytosolic LDH in cancer cells could potentially decrease several classes of cancer proliferation rates, including pancreatic tumors, renal cell carcinoma, bladder cancer, and nonsmall-cell lung cancer (NSCLC) (192). LDH gene expression can be upregulated epigenetically (methylation, acetylation, lactylation), transcriptionally, and post-translationally. It was recently demonstrated that incubating H1299 (nonsmall cell lung cancer) cells with lactate resulted in downregulation of enzymes supporting glycolytic flux (hexo- and pyruvate kinases), while enzymes of oxidative metabolism (isocitrate and succinate dehydrogenases) were upregulated (193). Because the authors also observed increased levels of histone lactylation, there may be a connection between this epigenetic modification and changes in the entire metabolic pathway. The myriad of modifications to LDH can promote a range of malignant phenotypes via cell proliferation, survival, metastasis, oxidative stress protection, and angiogenesis induction, thereby supporting persistent growth (194). As a biomarker, serum levels of LDH can serve as an index in cancer diagnosis (192). Likewise, a noteworthy procedure revealed that surgical removal of tumors resulted in decreased levels of serum LDH (195). With such a diverse set of factors that can influence cancer cell survival, decreasing LDH activity via silencing may serve as a therapeutic treatment. For example, the silencing LDH in transgenic NSCLC mouse models has shown to decrease tumorigenesis and disease curtailment after 6 wk of gene knockout (196). Furthermore, the use of potassium oxamate, an LDH inhibitor, has been shown to also decrease lactate production and may be a promising anticancer agent in human gastric cancer cells (197) and HeLa cells in tissue culture (198). Moreover, clinical trials using gossypol, a cotton plant-derived phenol, is known to compete with NADH and possesses anticancer effects in vivo. When administered orally, adrenal tumor size was reduced (199) and in patients with metastatic breast cancer, serum tumor markers were decreased (200).

In closing this section, it is appropriate to comment on the seemingly contrasting roles of lactate in encouraging a healthy phenotype while also being involved in carcinogenesis. To reiterate, endurance training and cancer phenotypes have a lot in common, including the presence of high glycolytic rates, resulting in lactate production and accumulation (9). Indeed, high rates of glucose consumption and lactate production are hallmarks of cancer, the so called Warburg Effect (201). Accordingly, it is a concern that lactatemia resulting from high-intensity interval training (HIIT) could induce transformation of cancer-prone cells. However, results of epidemiological studies support the idea that regular physical activity reduces the risk of many common cancers, including cancer of the breast, colon, bladder, uterus, esophagus, kidney, lung, and stomach. It is noteworthy that the organs protected from cancer by physical exercise have apparently little to do with exercise itself, suggesting the presence of a protective cytokine, myokine, adipokine, or metabolite during exercise (202). Given this observation, a proposal is that intermittent lactate release and circulation during physical activity improves lactate clearance and preconditions cells, tissues, and organs by reducing the chance that lactagenesis promotes carcinogenesis (9).

Recently, Feng et al. (59) may have provided a mechanistic explanation of the “exercise prevents/lactate promotes cancer” dichotomy. Using a mouse model with transplanted MC38 tumors the investigators found that subcutaneous administration of sodium lactate resulted in CD8+ T cell-dependent tumor growth inhibition. Single cell transcriptomics analysis revealed increased proportion of stem-like TCF-1-expressing CD8+ T cells among intratumoral CD3+ cells. Their results indicated that exogenous lactate inhibits histone deacetylase activity, which resulted in increased acetylation at H3K27 of the TCF7 super enhancer locus, ultimately increasing TCF7 gene expression. As well, the investigators showed that CD8+ T cells pretreated with lactate efficiently inhibited tumor growth when transferred to tumor-laden mice. Consequently, the investigators interpreted their results to mean that sodium lactate could provide tumor immunity. Interestingly, glucose did not have a similar effect. This is important because in tumors the low pH environment retards protective effects of CD8+ T cells.

As exciting as the results appear, it is clearly early-stages in terms of proposing lactate infusion as cancer immune therapy. One consideration is that physical exercise raises both lactate anion and hydrogen ion concentrations. In contrast, sodium lactate administration results in a mild alkalosis (203). Hence, it could be that the alkalosis of sodium or other, nonacidic lactate compounds could mitigate the effects of low-pH environments, thus facilitating the protective effects of CD8+ T cells. Using lactate anions to mitigate the effects of acidosis and provide nutritional support in exercise (18) and sepsis (204) is not new. Hopefully, in the near future new technologies such as fluorescent indicators of lactate (FiLa) (205) will advance our understanding of the role of lactate in health and disease.

The role of lactate in cancer biology is a huge field worthy of a volume of reviews. Suffice it to reassert that lactate upregulates a glycolytic cell phenotype while also suppressing an oxidative phenotype. Lactate also supports angiogenesis (58), cell migration, metastasis, and self-sufficient metabolism, all of which encourage progression to cancer (3, 9).

#### Lactate and other maladies.

Studies on cultured osteoclasts indicate that glycolysis leads to lactate production and that lactate is the active metabolite mediating bone resorption (62). As such, investigators are exploring ways to block glycolysis and lactate production in osteoclasts as a therapeutic strategy in diseases characterized by osteoclast-mediated bone loss such as ovariectomy, postmenopausal osteoporosis, and rheumatoid arthritis.

#### Lactate signaling and sensing in mimicking glucose resulting in hyperinsulinemia and hypoglycemia.

Lactate-glucose interactions are complex, but usually glucose, not lactate controls insulin secretion. However, problems can arise if lactate interferes with glucose-insulin signaling. Classically, as recognized in Cori cycle (60) and the lactate shuttle (1, 70), glucose and glycogen are the precursors to lactate formation (2, 206), and lactate is the major gluconeogenic precursor (60, 207–210). However, whereas blood glucose levels provide important feedback in the regulation of insulin and counter-regulatory hormones, lactate normally plays no direct role in the regulation of insulin secretion and by that mechanism lactate is excluded from the regulatory processes.

In the normal pancreatic islet, MCT gene expression is silenced, and hence protein synthesis and insertion into β-cell plasma membranes is prevented (211, 212). The silencing of MCT expression in pancreatic β-cells keeps extracellular lactate from affecting intracellular redox and thereby interfering with glucose sensing and insulin secretion (213). Silencing of MCT1 in pancreatic β-cells is evolutionary proof that lactate overrides glucose in regulating energy substrate partitioning in general, and insulin secretion in particular when the dominant role of lactate must be suppressed. In this regard, it is noteworthy that persons with failed silencing of MCT1 expression and resulting MCT insertion into plasma membranes of pancreatic β-cells become hypoglycemic during hard exercise. This is because the presence of plasma membrane MCT1 allows lactate to gain entry into pancreatic β-cells that affects cell redox, just as if blood glucose was elevated. Thus, the signal is misinterpreted as indicating systemic hyperglycemia (that does not exist), thereby stimulating pancreatic insulin secretion, and increased glucose disposal causing hypoglycemia (214).

### HIF and/or LIF?

The transcription factor hypoxia inducible factor-1 (HIF-1) is recognized for being the master regulator of oxygen homeostasis (215). Knowledge of its role in exercise was inspired by results from studies of cell biology, including cancer biology, rodent and human studies (216). Literature on the subject HIF-1 expression shows a tight relationship with glycolysis such that one is tempted to consider thinking of the transcription factor also as a “lactate induced factor” (LIF), particularly if feedback control of HIF-1 is considered. This association has been previously mentioned (3) and described more fully (217), but remains unclear (218, 219). For the present, the hypoxia inducible/lactate induced factor (HIF/LIF) appears to have both direct signaling and indirect physiological effects resulting in a more glycolytic, and less oxidative muscle phenotype. Exceptions may include VEGF formation (46) and upregulation of MCT expression (220). Consequently, from the standpoint of using regular physical exercise to maintain or improve health over the lifespan (15, 218, 221), at present the role of HIF in adaptation to exercise is not completely understood.

HIF-1 is a heterodimeric molecule with pairs of two subunits: HIF-1α (regulatable subunits) and HIF-1β (constitutively expressed), dimers with a purported evolutionary role in high altitude adaptation (222). HIF-1β is also termed the aryl hydrocarbon receptor nuclear translocator (ARNT). After synthesis, HIF-1α is hydroxylated on proline residues by prolyl hydroxylase 1–3 (PHD1-3). This allows for ubiquitination by the von Hippel–Lindau ubiquitin ligase E3 (VHL E3), leading to degradation of the protein complex by the 26s proteasome. During hypoxia (low oxygen concentration), PHD1-3 is inhibited and HIF-1α is not degraded and remains active (223). In cancer cells a similar effect of lactate in activating HIF-1 was first observed (224–226).

Consistent with the concept that HIF promotes a glycolytic phenotype, constitutively in mice HIF-1 is higher in fast than slow twitch muscles and is increased following high-intensity exercise training (227). HIF-1 increases gene and protein expression of pyruvate dehydrogenase kinase (PDHK), thus phosphorylating and inactivating the PDH complex which is responsible for catalyzing the decarboxylation of pyruvate to acetyl-coenzyme A, the first step in the mitochondrial catabolism of pyruvate (228). As a consequence, by increasing expression of PDHK HIF acts to downregulate oxidative metabolism, decrease lactate clearance, and promote lactate accumulation, which are not desirable effects for health, healthy aging, or exercise endurance.

Data on HIF expression and signaling by oxygen and high lactate obtained on studies using cell culture techniques and rodent models need to be understood by comparison with results of studies on humans. Studies on normoxic humans show that the intramuscular partial pressure of oxygen (Po_2_) remains above the critical mitochondrial Po_2_ during exercise eliciting maximal oxygen uptake (V̇o_2max_) (229). Moreover, in a clever, one leg knee extensor training study Lundby et al. demonstrated a short-term (6-h) effect of exercise in HIF-1α and -2α expression that was attenuated by exercise training. Because exercise testing and training studies were conducted under normoxia and neither muscle Po_2_ or lactate levels were measured, the authors concluded the changes in HIF expression were exercise, but not hypoxia-induced (230). More recently to assess the effects of high-intensity interval training (HIIT) on muscle gene expression, Norrbom et al. used cutting-edge Transcription-Factor Motif-Enrichment Analyses on leg muscle from 11 men before and after nine bouts of HIIT, (3 times/wk) (3 wk) (219). They found that almost 2,000 genes across 84 pathways were differentially expressed in response to a single HIIT session. Most prominent among those was upregulation of HIF-1α expression. Overall, the transcriptional response to acute exercise was strikingly similar at 3 wk, 83% (*n* = 1,650) of the genes regulated after the 1st compared with the ninth bout. Again, neither muscle Po_2_ nor lactate levels were measured (219). However, as seen previously (230), the responses post-training were 30% attenuated compared with the first bout. The attenuation differed substantially between pathways and was especially pronounced for glycolysis and cellular adhesion compared with MAPK pathway genes such as that coding for VEGF.

At present, it is appropriate to suspect that the HIF low oxygen/high lactate response is part of the transient response to exercise training, particularly with regard to the glycolytic aspects of muscle metabolism. However, HIF-related effects observed in cell systems and in mammalian models are to be considered along with results from a plethora of studies showing that endurance training increases cardiovascular capacity in women and men (231–233), increases muscle perfusion (234), stimulates mitochondrial biogenesis (32, 93, 94), increases the expression of monocarboxylate transporter isoform 1 (MCT1) and subtly shifts the pattern of LDH A/B expression (218, 220, 235), and increases lactate clearance (76, 236). Hence, it appears that some, but not all of the outcomes of HIF-1 signaling occur in humans during exercise or as a consequence of exercise training (218, 219).

### Does the Future Stem from the Beginning?

As articulated at the outset, to date literature on lactate signaling and sensing fall largely within the domains of exercise and nutrient delivery metabolism. However, as investigators turn the page and delve into new areas of lactate biology we will better understand how perturbations in lactate turnover and accumulation, sensing and signaling could have beneficial or other, sometimes detrimental, consequences. For instance, Rinaudo and coworkers (237) showed that the hyperoxic environment of in vitro fertilization can result in perturbations in the L/P and ROS generation that are associated with insulin resistance and loss of metabolic flexibility in offspring (238). In concert, it has been shown that pyruvate is indispensable for preimplantation development and zygotic gene activation (ZGA) beyond 2-cell (2C) stage of development, following which either pyruvate or lactate can facilitate continued cell development and ZGA (239–241). In contrast, neither glucose nor glutamine was able to advance development and ZGA beyond 2C (17). Of particular interest is histone lactylation, not only for the effects of gene expression in adults, but also in early stages of development (242).

Beyond the possibility that lactate could influence nuclear gene expression by lactylation, another emerging possibility is that lactate could influence the mitochondrial genome. The mitochondrial reticulum contains multiple copies of a distinct circular genome containing 13 protein-encoding genes. However, short open reading frames (sORFs) encoded in the mitochondrial genome have been recently identified. Importantly, such sORFs produce bioactive peptides, collectively referred to as mitochondrial-derived peptides (MDPs), which have broad physiological functions (243, 244). MOTS-c (mitochondrial ORF of the 12S rRNA type-c) is an MDP that is purported to promote “metabolic homeostasis” in response to stress. Consequently, MOTS-c has been referred to a “mitokine.” At present regulation of MOTS-c expression appears to be under dual control, in part, via AMPK (245), and in part by ROS (244) that determine the adaptive nuclear gene expression following nuclear translocation (246).

In a recent study examining the role of exercise on ameliorating the effects of aging on muscle metabolic homeostasis, Reynolds et al. (247) gave MOTS-c to mice and cultured myocytes and determined the MOTS-c response in exercise humans. Balloon plots derived from RNA-seq data of MOTS-c treated skeletal muscle from old mice showed activation of AMPK. In addition, C2C12 myoblasts showed common transcription factors, including those influencing the response to oxidative stress, protein localization to the nucleus, and mitochondrial organization. Despite the operating hypothesis that MOTS-c is involved in preservation of cellular metabolic homeostasis in response to stress, the authors failed to measure lactate in cultured myocytes, exercised mice or humans. The myoblast response to lactate includes AMPK activation and ROS generation (32). Hence, could it be that an exerkine (lactate) gives rise to a mitokine (MOTS-c)? This potential role of lactate signaling in promoting “metabolic homeostasis” warrants further investigation.

## CONCLUSIONS

The role of lactate in normal and pathological conditions has come a long way from its traditional view as a metabolic waste product and cause of muscle fatigue (1–6). Lactate works in diverse ways to affect physiology and metabolism; sometimes the action is direct such as in the lactate receptor HCAR-1, or other times in concert with other signals such as via with the carotid body olfactory receptor (Olfr78) in the control of breathing. Certainly, lactate is not the only myokine of exerkine (15), but lactate has important signaling functions to be considered. In terms of energy substrate partitioning lactate is at the fulcrum of metabolic regulation, at low levels either permissive of lipolysis and mitochondrial fatty acid oxidation, or at high levels inhibiting lipolysis and mitochondrial fatty acid uptake and oxidation (13). Lactate is formed under fully aerobic conditions during postprandial rest and exercise (4, 10, 14). As revealed by the presence of the postprandial lactate shuttle (4), lactate is the metabolic intermediate involved in dietary carbohydrate distribution and disposal. Mechanisms by which lactate operates to control energy substrate partitioning include mass action (3), allosteric binding (23, 24, 248), ROS production (32), canonical intracellular signaling (249), central nervous system signaling via substrate supply (153) and protein lactylation (148), and gene expression via histone lactylation (30, 250). With all due respect to classical and contemporary discoveries in metabolic regulation, it is reasonable to assert that “lactate is the major myokine and exerkine” because of its abundance, dynamic range of concentration change, effect on cell redox and multiple independent and coordinated regulatory effects on major metabolic pathways in diverse tissues (115, 251). Lactate fuels the spiral mitochondrial reticulum at the base of the sperm head. The event of conception is followed by the influence of lactate on embryonic development (17, 242), and subsequently over the lifespan (238).

## GRANTS

This work was supported by National Institutes of Health (NIH) grant no. R01 AG059715-01 and the UCB Center for Research and Education on Aging (CREA) (to G. A. Brooks).

## DISCLOSURES

No conflicts of interest, financial or otherwise, are declared by the authors.

## AUTHOR CONTRIBUTIONS

G.A.B. conceived and designed research; G.A.B. performed experiments; G.A.B., A.D.O., J.A.A., J.J.D., and R.G.L. analyzed data; G.A.B., A.D.O., J.A.A., J.J.D., C.C.C., D.D.M.-S., and R.G.L. interpreted results of experiments; G.A.B., J.A.A., C.C.C., and D.D.M.-S. prepared figures; G.A.B. drafted manuscript; G.A.B., A.D.O., J.A.A., J.J.D., C.C.C., D.D.M.-S., and R.G.L. edited and revised manuscript; G.A.B., A.D.O., J.A.A., J.J.D., C.C.C., D.D.M.-S., and R.G.L. approved final version of manuscript.

## Footnotes

1 PubMed searches on October 4, 2022 for V̇o_2max_ produced 1,187 hits, whereas exercise lactate produced 1,734 hits.

2 Readers are referred to https://www.broadinstitute.org/scientific-community/science/programs/metabolic-disease-program/publications/mitocarta/mitocarta-in-0 (MitoCarta) and http://mitominer.mrc-mbu.cam.ac.uk/release-4.0/begin.do (MitoMiner).
